# Crystal structure and Hirshfeld surface analysis of luteolin dimethyl sulfoxide monosolvate

**DOI:** 10.1107/S2056989026002720

**Published:** 2026-03-19

**Authors:** Jia Xu

**Affiliations:** a School of Pharmacy, Jiangsu Medical College, Yancheng 224005, People’s Republic of China; Illinois State University, USA

**Keywords:** luteolin, dimethyl sulfoxide, Hirshfeld surface analysis, crystal structure

## Abstract

The title compound, 3′,4′,5,7-tetrahydroxyflavone dimethyl sulfoxide monosolvate (LUT-DMSO), C_2_H_6_OS·C_15_H_10_O_6_, crystallizes in the monoclinic space group *P*2_1_. The LUT mol­ecule adopts a planar conformation, and the crystal structure is consolidated by extensive hydrogen-bonding inter­actions. Hirshfeld surface analysis indicates that the predominant inter­molecular inter­actions are O⋯H/H⋯O, C⋯H/H⋯C, and H⋯H, with these contacts contributing significantly to the overall cohesion of the crystal.

## Abstract 2

The title compound crystallizes in the monoclinic space group *P*2_1_. The luteolin mol­ecule adopts a planar conformation, and the crystal structure is consolidated by extensive hydrogen-bonding inter­actions.

## 1. Chemical context

Luteolin (3′,4′,5-tetra­hydroxy­flavone) is a naturally occurring flavonoid found in multiple flora such as honeysuckle, scutellaria (Lamiaceae), dandelion (Asteraceae), as well as peanut shells and corn whiskers (Mahwish *et al.*, 2025 ▸). This compound has received worldwide research inter­est due to its wide variety of biological activities, particularly anti­oxidant, anti-inflammatory, anti­cancer, and neuroprotective properties (Zhang & Ma, 2024 ▸). Luteolin is a potent anti­oxidant which promotes the expression of anti­oxidant enzymes (*e.g.* SOD, heme oxygenase-1, HO-1) and scavenges reactive oxygen species (ROS) therefore reducing oxidative stress. Luteolin’s anti-inflammatory activity results from the inhibition of numerous pro-inflammatory cytokines and enzymes (TNF-*α*, IL-6, COX-2, iNOS) and the modulation of cellular signalling pathways (NF-*κ*B, MAPK/AP-1) (Pandey *et al.*, 2025 ▸). With regards to cancer, luteolin has been shown to induce apoptosis, inhibit cell proliferation, and reduce angiogenesis in various cancer models from breast, colon, and pancreatic cancer (Prasher *et al.*, 2022 ▸). Research has shown that luteolin exerts neuroprotection in models of Alzheimer’s and Parkinson’s disease by decreasing neuro-inflammation, oxidative damage, and neuronal apoptosis (Zhu *et al.*, 2024 ▸).
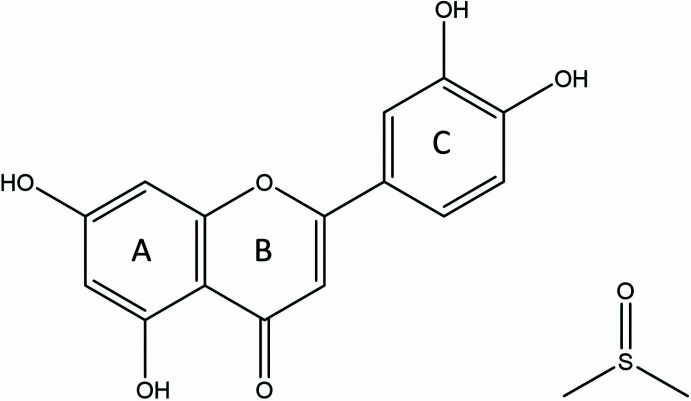


In this study, we report the crystal structure and Hirshfeld surface analysis of luteolin dimethyl sulfoxide solvate, which has not previously been reported in the literature.

## 2. Structural commentary

**Figure 1:**
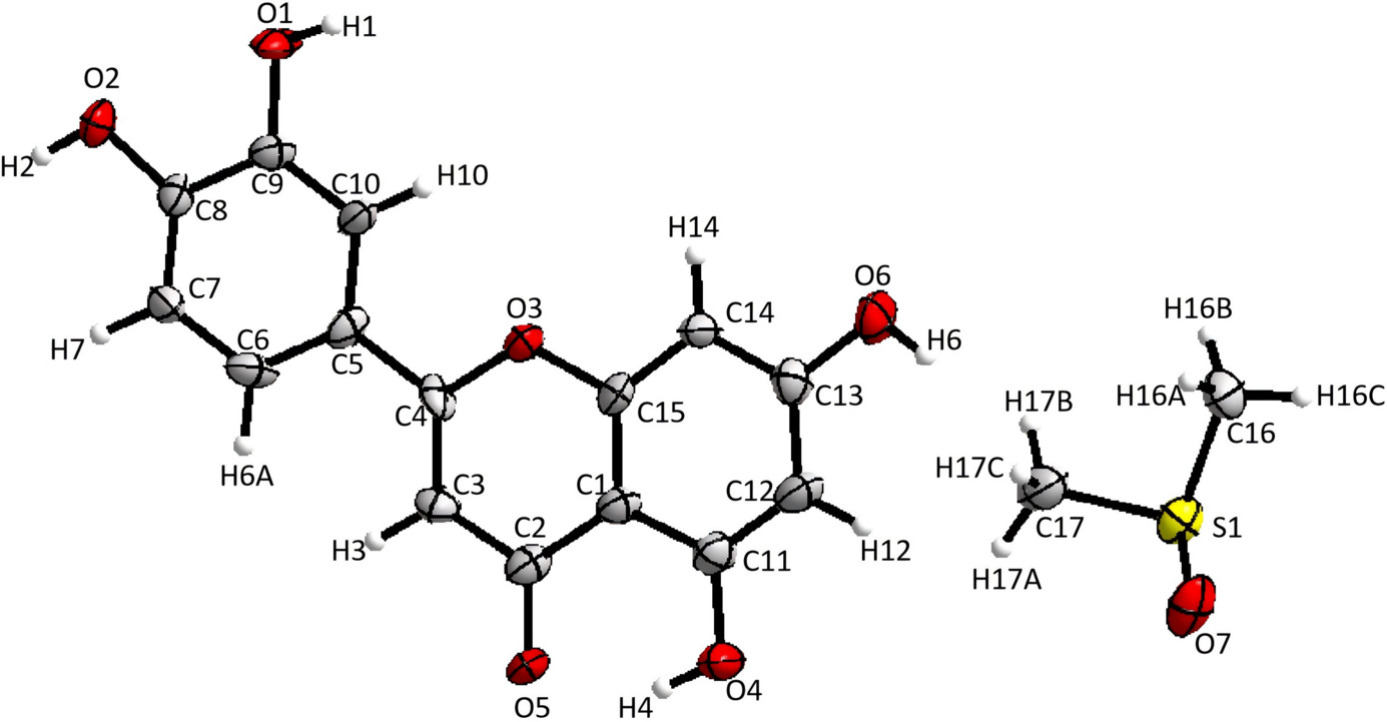
The mol­ecular structure of LUT-DMSO, with atomic displacement ellipsoids drawn at the 30% probability level, showing the atom labeling. Hydrogen atoms are represented as small spheres with arbitrary radii.

LUT-DMSO crystallizes in the monoclinic space group *P*2_1_. The asymmetric unit consists of one LUT mol­ecule and one DMSO mol­ecule, as depicted in Fig. 1 ▸. The luteolin mol­ecules adopt a planar configuration due to conjugation in the flavonoid backbone. The dihedral angle between the fused rings is 2.2 (5)° and that between the phenyl ring and the fused ring system is 3.1 (4)°. The torsion angles for O3—C4—C5—C6, O3—C4—C5—C10, C3—C4—C5—C6, and C3—C4—C5–C10 are 177.7 (9), −1.9 (14), −1.8 (16), and 178.6 (10)°, respectively. Several intra­molecular hydrogen bonds are also observed within the LUT mol­ecules (O4—H4⋯O5, O6—H6⋯O7, and C10—H10⋯O3; Table 1 ▸).

## 3. Supra­molecular features

**Figure 2:**
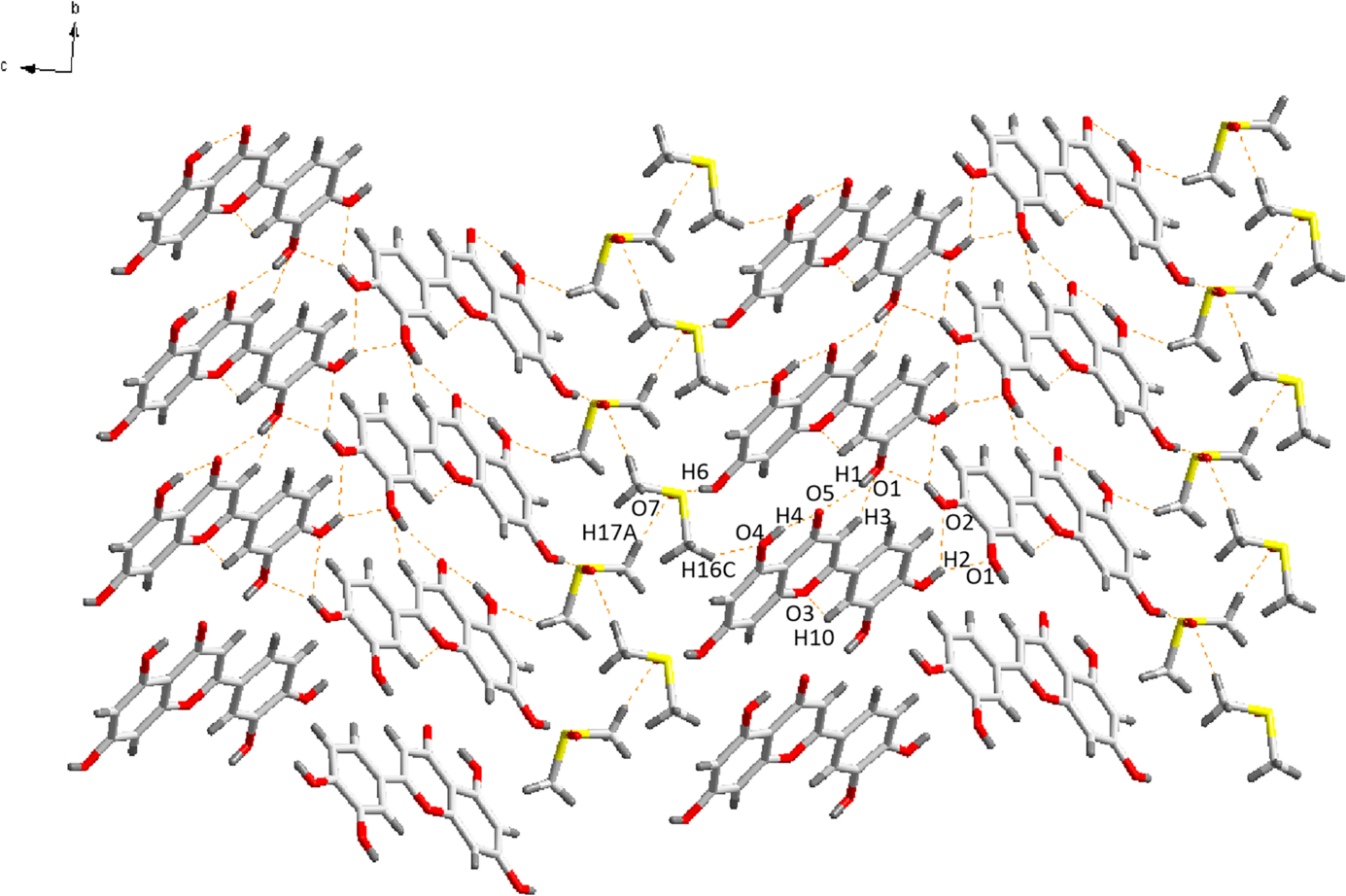
Hydrogen-bond networks in LUT-DMSO, showing the two-dimensional network parallel to the (100) plane (hydrogen bonding is indicated by orange dashed lines).

In the crystal, each LUT and DMSO mol­ecule is involved in extensive hydrogen-bonding inter­actions, contributing to crystal cohesion (Fig. 2 ▸, Table 1 ▸). DMSO mol­ecules are connected to neighboring DMSO mol­ecules through C17—H17*A*⋯O7 inter­actions, forming a chain-like structure. The DMSO mol­ecules also inter­act with LUT mol­ecules through C16—H16*C*⋯O4 and O6—H6⋯O7 hydrogen bonds. The LUT mol­ecules are connected to each other through O1—H1⋯O5, C3—H3⋯O1, O2—H2⋯O1, and O2—H2⋯O2 hydrogen bonds. Collectively, these interactions give rise to a two-dimensional network that lies parallel to the (100) plane, as illustrated in Fig. 2 ▸.

To validate the hydrogen-bonding inter­actions observed in LUT-DMSO, we have compared the observed donor–acceptor distances with those found in other luteolin-containing crystal structures, such as ZIKPUG01 and ZIKPUG02 (cocrystals with isonicotinamide; Sowa *et al.*, 2013 ▸) and VOHKIO (a dapsone-luteolin-ethanol solvate; Jiang *et al.*, 2014 ▸). In our structure, the intra­molecular O⋯O distances are 2.626 (10) Å (O4—H4⋯O5) and the C⋯O distance is 2.682 (11) Å (C10—H10⋯O3). The inter­molecular O⋯O distances range from 2.649 (11) to 2.989 (11) Å, and the inter­molecular C⋯O distances range from 3.220 (13) to 3.308 (18) Å. When compared with other luteolin-containing crystal structures, all *D*⋯*A* distances in the title structure fall within typical ranges for O—H⋯O and C—H⋯O hydrogen bonds, except for the intra­molecular C⋯O contact (C10—H10⋯O3), which appears slightly shorter. However, this short distance is likely due to the rigidity of the luteolin backbone, which forces the O3 and C10 atoms into close proximity, rather than an artefact of the riding model. The H⋯*A* distance of 2.35 Å for this inter­action is well within the range of normal C—H⋯O hydrogen bonds. No similar intra­molecular C—H⋯O hydrogen bond has been reported in other LUT structures, possibly because of differences in mol­ecular conformation or crystal packing. Therefore, we conclude that the hydrogen-bonding geometry in the title structure is reasonable and consistent with known LUT-containing crystals.

## 4. Hirshfeld surface analysis

**Figure 3:**
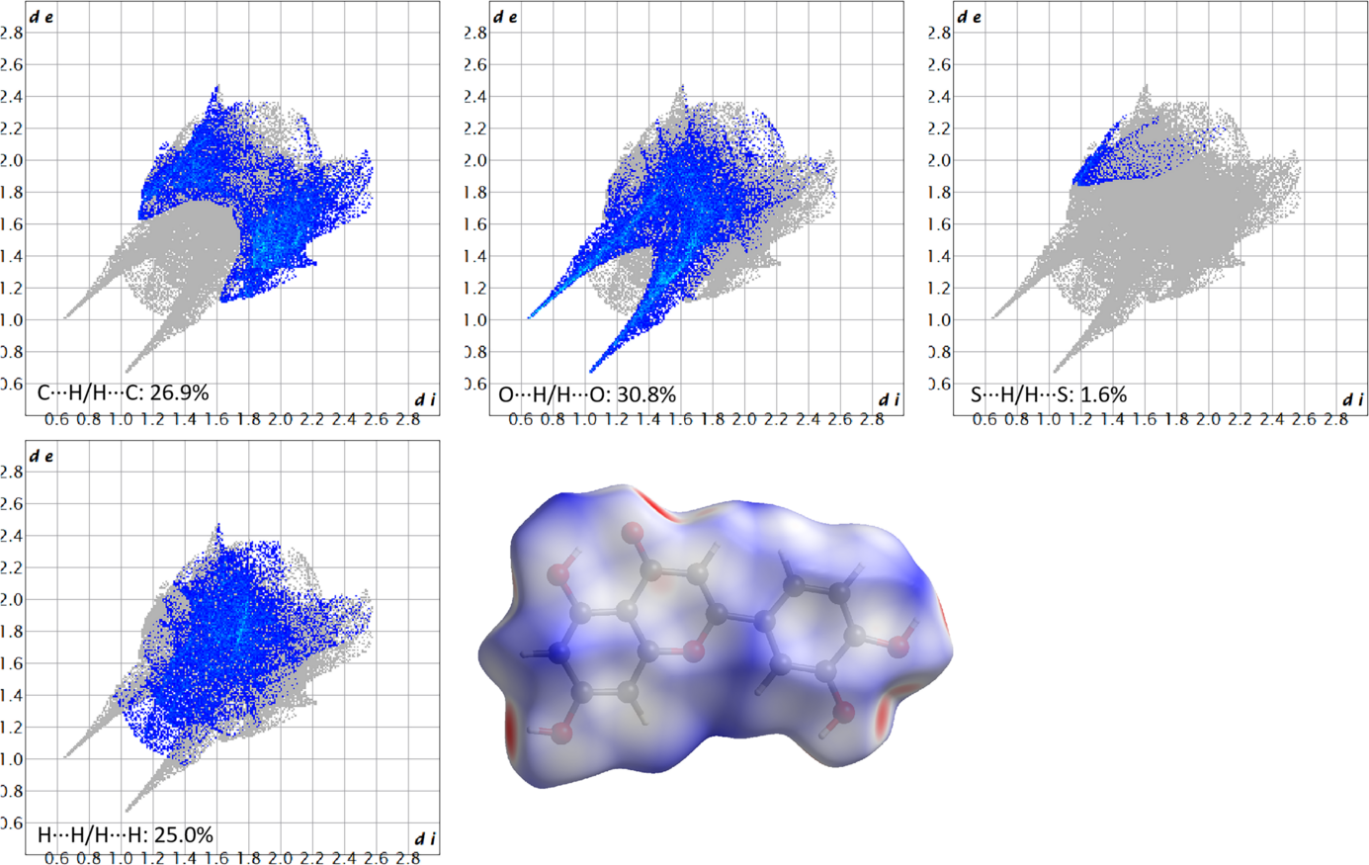
Hirshfeld surfaces and the corresponding two-dimensional fingerprint plots of various hydrogen-bonding and van der Waals inter­actions for the LUT mol­ecule of LUT-DMSO. The *d*_i_ and *d*_e_ values represent the closest inter­nal and external distances (in Å), respectively, from given points on the Hirshfeld surface.

**Figure 4:**
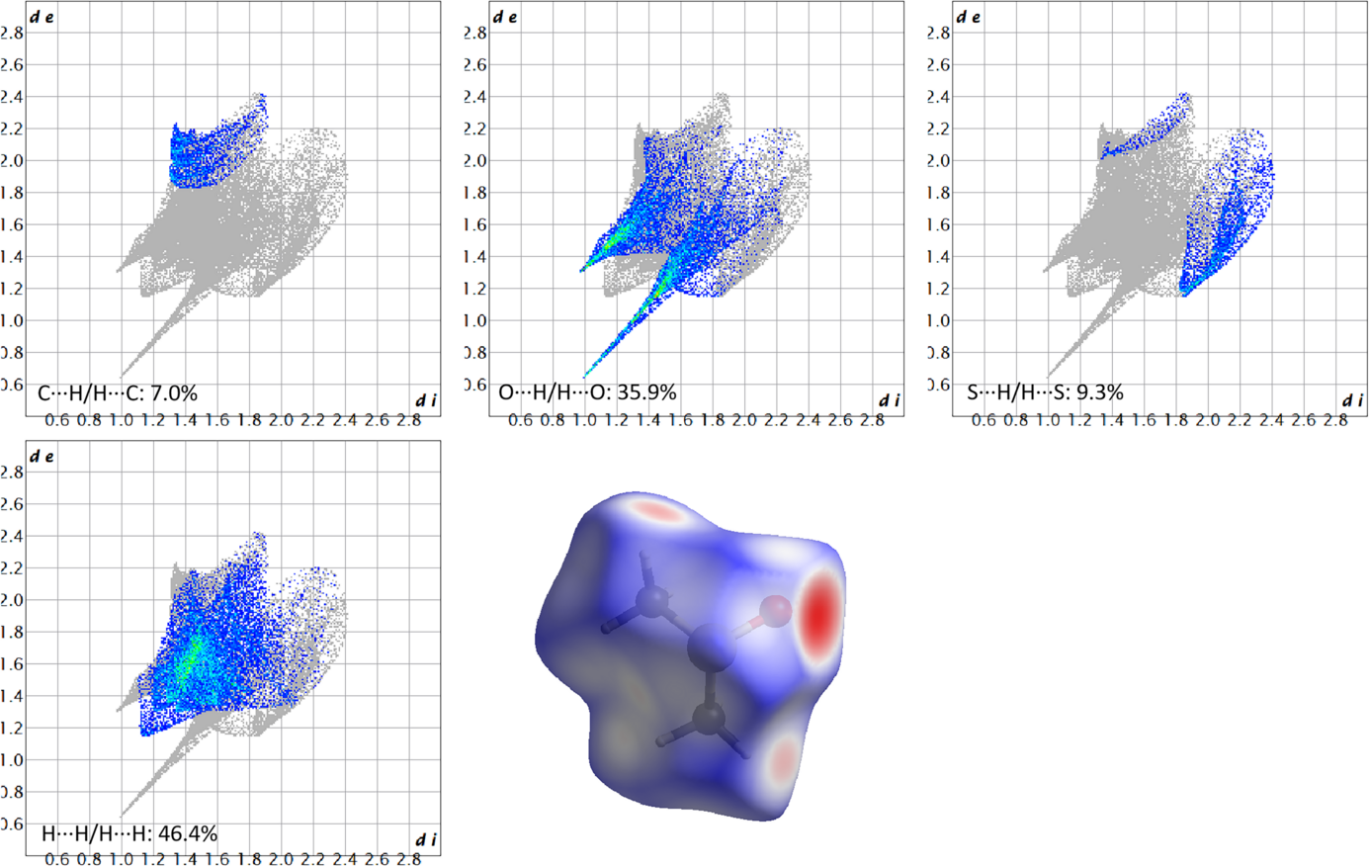
Hirshfeld surfaces and the corresponding two-dimensional fingerprint plots of various hydrogen-bonding and van der Waals inter­actions for the DMSO mol­ecule of LUT-DMSO. The *d*_i_ and *d*_e_ values represent the closest inter­nal and external distances (in Å), respectively, from given points on the Hirshfeld surface.

The Hirshfeld surface analysis of LUT-DMSO was conducted to evaluate the inter­molecular inter­actions within the crystal structure. Hirshfeld surfaces and fingerprint plots (Spackman & McKinnon, 2002 ▸; Spackman & Jayatilaka, 2009 ▸) were generated using *CrystalExplorer* software (Spackman *et al.*, 2021 ▸). Fig. 3 ▸ shows the Hirshfeld surfaces and the corresponding two-dimensional fingerprint plots for the LUT mol­ecule, while Fig. 4 ▸ illustrates the same for the DMSO mol­ecule.

For the LUT mol­ecule, the predominant inter­molecular inter­actions are O⋯H/H⋯O, C⋯H/H⋯C, H⋯H, and S⋯H/H⋯S, which account for 84.3% of the total inter­actions, indicating their significant role in consolidating the structure. Among these, O⋯H/H⋯O inter­actions are the most prevalent, contributing 30.8% of the Hirshfeld surface, followed by C⋯H/H⋯C (26.9%) and H⋯H (25.0%) inter­actions. The S⋯H/H⋯S inter­action is relatively weak, contributing only 1.6% of the Hirshfeld surface.

For the DMSO mol­ecule (Fig. 4 ▸), the most significant contacts are H⋯H, O⋯H/H⋯O, S⋯H/H⋯S and C⋯H/H⋯C. The H⋯H inter­action is the dominant contributor, accounting for 46.4%, followed by O⋯H/H⋯O (35.9%), S⋯H/H⋯S (9.3%) and C⋯H/H⋯C (7.0%) inter­actions. The total contribution of these inter­actions is 98.6%, emphasizing their dominant role in the structural cohesion.

In both mol­ecules, the Hirshfeld surface offers a clear depiction of mol­ecular inter­actions, with the majority of red spots on the surface corresponding to O⋯H inter­actions, which are also reflected in the 2D fingerprint plots as prominent spikes. These findings emphasize the crucial role of hydrogen bonding and van der Waals inter­actions in determining the packing and cohesion of the LUT-DMSO crystal.

## 5. Database survey

A survey of the Cambridge Structural Database WebCSD, May 2025; Groom *et al.*, 2016 ▸) did not reveal any structures of LUT-DMSO. The survey revealed seven crystal structures that related to LUT compounds, *viz*. OJEQUP, EJEPUG, EJEQIV, VOHKIO, ZIKPUG, ZIKPUG01 and ZIKPUG02. OJEQUP (Cox *et al.*, 2003 ▸) is a hemihydrate of luteolin, although the water mol­ecules were disordered and not located, which crystallize in the monoclinic *C*2 space group. EJEPUG and EJEQIV (He *et al.*, 2016 ▸) are cocrystals of LUT with proline. Luteolin is a poorly soluble compound and the cocrystallization of LUT with l-proline and d-proline is useful for solubility enhancement. Additionally, the structures VOHKIO (a dapsone-luteolin ethanol solvate; Jiang *et al.*, 2014 ▸) and ZIKPUG, ZIKPUG01, ZIKPUG02 (cocrystals of luteolin with isonicotinamide, with ZIKPUG and ZIKPUG01 representing one polymorph and ZIKPUG02 a second polymorph; Sowa *et al.*, 2013 ▸) have also been reported, further illustrating the versatility of luteolin in forming multicomponent crystals and its polymorphic behavior.

## 6. Synthesis and crystallization

The commercially available form of luteolin (98%) was purchased from Aladdin. LUT (50 mg, 0.18 mmol) was dissolved in 15 mL of DMSO by heating. Colorless plate-like single crystals were obtained by slowly evaporating the filtrated solution at room temperature for 30 days.

## 7. Refinement

Crystal data, data collection and structure refinement details are summarized in Table 2 ▸. Due to the limited data quality, the hydrogen atoms attached to oxygen atoms could not be located in the difference-Fourier map. Therefore, all H atoms were placed in geometrically calculated positions and refined using a riding model.

## Supplementary Material

Crystal structure: contains datablock(s) I. DOI: 10.1107/S2056989026002720/ej2014sup1.cif

Structure factors: contains datablock(s) I. DOI: 10.1107/S2056989026002720/ej2014Isup5.hkl

Supporting information file. DOI: 10.1107/S2056989026002720/ej2014Isup3.cml

CCDC reference: 2537224

Additional supporting information:  crystallographic information; 3D view; checkCIF report

## Figures and Tables

**Table 1:** Hydrogen-bond geometry (Å, °)

*D*—H⋯*A*	*D*—H	H⋯*A*	*D*⋯*A*	*D*—H⋯*A*
O1—H1⋯O5^i^	0.84	1.83	2.649 (11)	163
O2—H2⋯O1^ii^	0.84	2.14	2.846 (11)	142
O2—H2⋯O2^ii^	0.84	2.35	2.989 (11)	133
O4—H4⋯O5	0.84	1.79	2.626 (10)	170
O6—H6⋯O7^iii^	0.84	1.79	2.626 (10)	170
C3—H3⋯O1^iv^	0.95	2.54	3.220 (13)	129
C10—H10⋯O3	0.95	2.35	2.682 (11)	100
C16—H16*C*⋯O4^iii^	0.98	2.44	3.293 (15)	145
C17—H17*A*⋯O7^v^	0.98	2.39	3.308 (18)	156

Symmetry codes: (i) 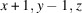; (ii) 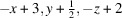; (iii) 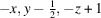; (iv) 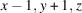; (v) 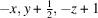.

**Table 2:** Experimental details

Crystal data	
Chemical formula	C_2_H_6_OS·C_15_H_10_O_6_
*M*_r_	364.36
Crystal system, space group	Monoclinic, *P*2_1_
Temperature (K)	170
*a*, *b*, *c* (Å)	6.676 (2), 5.6991 (17), 20.724 (6)
β (°)	90.749 (11)
*V* (Å^3^)	788.4 (4)
*Z*	2
Radiation type	Mo *K*α
μ (mm^−1^)	0.25
Crystal size (mm)	0.12 × 0.06 × 0.04

Data collection	
Diffractometer	Bruker APEXII CCD
Absorption correction	Multi-scan (*SADABS*; Krause *et al.*, 2015 ▸)
*T*_min_, *T*_max_	0.479, 0.745
No. of measured, independent and observed [*I* > 2σ(*I*)] reflections	5797, 3000, 1894
*R*_int_	0.083
(sin θ/λ)_max_ (Å^−1^)	0.628

Refinement	
*R*[*F*^2^ > 2σ(*F*^2^)], *wR*(*F*^2^), *S*	0.095, 0.284, 1.04
No. of reflections	3000
No. of parameters	232
No. of restraints	1
H-atom treatment	H-atom parameters constrained
Δρ_max_, Δρ_min_ (e Å^−3^)	1.08, −0.56
Absolute structure	Flack *x* determined using 528 quotients [(*I*^+^)−(*I*^−^)]/[(*I*^+^)+(*I*^−^)] (Parsons *et al.*, 2013 ▸)
Absolute structure parameter	0.3 (2)

Computer programs: *APEX5* (Bruker, 2019 ▸) and *SAINT* (Bruker, 2019 ▸), *SHELXT2014/5* (Sheldrick, 2015*a* ▸), *SHELXL2025/1* (Sheldrick, 2015*b* ▸), *OLEX2* (Dolomanov *et al.*, 2009 ▸), *DIAMOND* (Pennington, 1999 ▸) and *PLATON* (Spek, 2020 ▸).

## supplementary crystallographic information

### 3',4',5,7-Tetrahydroxyflavone dimethyl sulfoxide monosolvate Crystal data

**Table:**

C_2_H_6_OS·C_15_H_10_O_6_	*F*(000) = 380
*M**_r_* = 364.36	*D*_x_ = 1.535 Mg m^−^^3^
Monoclinic, *P*2_1_	Mo *K*α radiation, λ = 0.71073 Å
*a* = 6.676 (2) Å	Cell parameters from 1758 reflections
*b* = 5.6991 (17) Å	θ = 3.0–25.4°
*c* = 20.724 (6) Å	µ = 0.25 mm^−^^1^
β = 90.749 (11)°	*T* = 170 K
*V* = 788.4 (4) Å^3^	Plate, colourless
*Z* = 2	0.12 × 0.06 × 0.04 mm

### 3',4',5,7-Tetrahydroxyflavone dimethyl sulfoxide monosolvate Data collection

**Table:**

Bruker APEXII CCD diffractometer	1894 reflections with *I* > 2σ(*I*)
φ and ω scans	*R*_int_ = 0.083
Absorption correction: multi-scan (SADABS; Krause *et al.*, 2015)	θ_max_ = 26.5°, θ_min_ = 2.0°
*T*_min_ = 0.479, *T*_max_ = 0.745	*h* = −7→8
5797 measured reflections	*k* = −7→7
3000 independent reflections	*l* = −25→25

### 3',4',5,7-Tetrahydroxyflavone dimethyl sulfoxide monosolvate Refinement

**Table:**

Refinement on *F*^2^	Hydrogen site location: inferred from neighbouring sites
Least-squares matrix: full	H-atom parameters constrained
*R*[*F*^2^ > 2σ(*F*^2^)] = 0.095	*w* = 1/[σ^2^(*F*_o_^2^) + (0.1706*P*)^2^] where *P* = (*F*_o_^2^ + 2*F*_c_^2^)/3
*wR*(*F*^2^) = 0.284	(Δ/σ)_max_ < 0.001
*S* = 1.04	Δρ_max_ = 1.08 e Å^−^^3^
3000 reflections	Δρ_min_ = −0.56 e Å^−^^3^
232 parameters	Absolute structure: Flack *x* determined using 528 quotients [(*I*^+^)-(*I*^-^)]/[(*I*^+^)+(*I*^-^)] (Parsons *et al.*, 2013)
1 restraint	Absolute structure parameter: 0.3 (2)
Primary atom site location: dual	

### 3',4',5,7-Tetrahydroxyflavone dimethyl sulfoxide monosolvate Special details

**Table:**

Geometry. All esds (except the esd in the dihedral angle between two l.s. planes) are estimated using the full covariance matrix. The cell esds are taken into account individually in the estimation of esds in distances, angles and torsion angles; correlations between esds in cell parameters are only used when they are defined by crystal symmetry. An approximate (isotropic) treatment of cell esds is used for estimating esds involving l.s. planes.

### 3',4',5,7-Tetrahydroxyflavone dimethyl sulfoxide monosolvate Fractional atomic coordinates and isotropic or equivalent isotropic displacement parameters (Å^2^)

**Table:**

	*x*	*y*	*z*	*U*_iso_*/*U*_eq_	
S1	0.1500 (4)	0.6686 (6)	0.42114 (13)	0.0341 (7)	
O3	0.7284 (9)	0.6422 (15)	0.7946 (3)	0.0290 (17)	
O5	0.2840 (9)	1.1285 (14)	0.8075 (3)	0.0307 (18)	
O2	1.4443 (10)	0.7383 (14)	0.9875 (4)	0.0338 (19)	
H2	1.455877	0.842373	1.015966	0.051*	
O1	1.3744 (10)	0.4339 (13)	0.8997 (4)	0.0344 (18)	
H1	1.328777	0.326151	0.876010	0.052*	
O6	0.3923 (11)	0.2151 (16)	0.6316 (4)	0.040 (2)	
H6	0.281819	0.208826	0.611833	0.060*	
O7	−0.0686 (9)	0.6616 (19)	0.4402 (3)	0.044 (2)	
O4	0.0868 (11)	0.9060 (15)	0.7183 (4)	0.039 (2)	
H4	0.110142	1.006989	0.746933	0.059*	
C8	1.2725 (15)	0.7742 (18)	0.9529 (5)	0.026 (2)	
C2	0.4218 (15)	0.9746 (19)	0.8028 (5)	0.029 (2)	
C3	0.5968 (16)	0.9797 (19)	0.8422 (5)	0.028 (2)	
H3	0.613272	1.103081	0.872606	0.034*	
C15	0.5642 (14)	0.6218 (19)	0.7539 (5)	0.028 (2)	
C1	0.4081 (15)	0.7865 (19)	0.7581 (5)	0.027 (2)	
C5	0.9248 (14)	0.8020 (18)	0.8774 (5)	0.026 (2)	
C6	0.9677 (15)	0.9718 (19)	0.9236 (5)	0.031 (2)	
H6A	0.877755	1.098973	0.929489	0.038*	
C9	1.2322 (14)	0.6052 (18)	0.9063 (5)	0.028 (2)	
C7	1.1419 (15)	0.957 (2)	0.9614 (5)	0.028 (2)	
H7	1.170055	1.074020	0.992951	0.033*	
C4	0.7407 (16)	0.8147 (17)	0.8377 (5)	0.027 (2)	
C10	1.0600 (14)	0.618 (2)	0.8688 (5)	0.032 (3)	
H10	1.033488	0.501847	0.836968	0.039*	
C14	0.5649 (15)	0.436 (2)	0.7119 (5)	0.030 (2)	
H14	0.676190	0.332721	0.709358	0.036*	
C11	0.2402 (15)	0.750 (2)	0.7166 (6)	0.033 (3)	
C13	0.3934 (16)	0.406 (2)	0.6728 (5)	0.032 (2)	
C17	0.2812 (16)	0.694 (3)	0.4955 (6)	0.043 (3)	
H17A	0.258720	0.850210	0.513707	0.065*	
H17B	0.424791	0.671107	0.488408	0.065*	
H17C	0.232927	0.574415	0.525507	0.065*	
C12	0.2344 (17)	0.565 (2)	0.6751 (6)	0.040 (3)	
H12	0.121274	0.543977	0.647397	0.048*	
C16	0.2299 (19)	0.368 (2)	0.4044 (6)	0.042 (3)	
H16A	0.194864	0.266227	0.440778	0.063*	
H16B	0.375277	0.364226	0.398410	0.063*	
H16C	0.162438	0.310979	0.365127	0.063*	

### 3',4',5,7-Tetrahydroxyflavone dimethyl sulfoxide monosolvate Atomic displacement parameters (Å^2^)

**Table:**

	*U*^11^	*U*^22^	*U*^33^	*U*^12^	*U*^13^	*U*^23^
S1	0.0286 (13)	0.0364 (15)	0.0373 (14)	−0.0047 (13)	−0.0010 (9)	0.0028 (14)
O3	0.020 (3)	0.037 (4)	0.030 (3)	0.004 (4)	−0.005 (2)	−0.005 (4)
O5	0.022 (3)	0.037 (5)	0.033 (4)	0.010 (3)	−0.008 (3)	−0.003 (4)
O2	0.026 (4)	0.040 (5)	0.035 (4)	−0.001 (3)	−0.012 (3)	−0.007 (3)
O1	0.029 (4)	0.018 (4)	0.055 (5)	0.009 (3)	−0.010 (3)	−0.008 (4)
O6	0.030 (4)	0.051 (6)	0.039 (4)	−0.006 (4)	−0.008 (3)	−0.009 (4)
O7	0.022 (4)	0.069 (6)	0.040 (4)	−0.015 (5)	−0.002 (3)	0.006 (5)
O4	0.032 (4)	0.036 (5)	0.051 (5)	0.013 (4)	−0.009 (3)	−0.011 (4)
C8	0.029 (5)	0.023 (5)	0.025 (5)	−0.002 (4)	−0.006 (4)	0.002 (5)
C2	0.024 (5)	0.024 (6)	0.037 (6)	−0.001 (4)	−0.005 (4)	0.004 (5)
C3	0.033 (6)	0.019 (5)	0.034 (6)	0.004 (4)	−0.001 (4)	0.000 (5)
C15	0.027 (5)	0.032 (6)	0.023 (5)	0.004 (5)	−0.007 (3)	0.002 (5)
C1	0.021 (5)	0.029 (6)	0.033 (6)	0.002 (4)	−0.002 (4)	0.001 (5)
C5	0.017 (5)	0.026 (6)	0.036 (6)	−0.005 (4)	−0.007 (4)	0.001 (5)
C6	0.021 (5)	0.024 (6)	0.049 (7)	0.003 (4)	0.005 (4)	−0.006 (5)
C9	0.021 (5)	0.028 (6)	0.036 (6)	0.003 (4)	0.000 (4)	0.000 (5)
C7	0.035 (6)	0.027 (6)	0.022 (5)	0.007 (5)	0.001 (4)	−0.003 (5)
C4	0.042 (6)	0.013 (5)	0.025 (6)	−0.004 (4)	−0.004 (4)	−0.005 (4)
C10	0.024 (5)	0.047 (7)	0.026 (5)	0.012 (5)	−0.004 (4)	0.001 (5)
C14	0.024 (5)	0.027 (6)	0.040 (6)	0.007 (4)	−0.006 (4)	−0.009 (5)
C11	0.025 (6)	0.034 (6)	0.038 (6)	0.001 (4)	−0.003 (4)	−0.003 (5)
C13	0.030 (6)	0.031 (6)	0.034 (6)	−0.004 (5)	−0.003 (4)	−0.006 (5)
C17	0.038 (6)	0.049 (8)	0.043 (6)	0.009 (6)	−0.002 (5)	0.006 (7)
C12	0.027 (6)	0.049 (7)	0.043 (7)	0.010 (5)	−0.007 (5)	0.002 (6)
C16	0.047 (7)	0.035 (7)	0.045 (7)	0.001 (6)	−0.009 (5)	−0.011 (6)

### 3',4',5,7-Tetrahydroxyflavone dimethyl sulfoxide monosolvate Geometric parameters (Å, º)

**Table:**

S1—O7	1.518 (7)	C15—C14	1.370 (15)
S1—C17	1.768 (12)	C1—C11	1.419 (15)
S1—C16	1.831 (13)	C5—C6	1.388 (15)
O3—C15	1.380 (11)	C5—C4	1.472 (14)
O3—C4	1.330 (12)	C5—C10	1.395 (14)
O5—C2	1.276 (12)	C6—H6A	0.9500
O2—H2	0.8400	C6—C7	1.397 (14)
O2—C8	1.359 (12)	C9—C10	1.382 (14)
O1—H1	0.8400	C7—H7	0.9500
O1—C9	1.370 (12)	C10—H10	0.9500
O6—H6	0.8400	C14—H14	0.9500
O6—C13	1.382 (14)	C14—C13	1.405 (14)
O4—H4	0.8400	C11—C12	1.362 (17)
O4—C11	1.356 (13)	C13—C12	1.398 (15)
C8—C9	1.389 (15)	C17—H17A	0.9800
C8—C7	1.372 (14)	C17—H17B	0.9800
C2—C3	1.416 (14)	C17—H17C	0.9800
C2—C1	1.420 (15)	C12—H12	0.9500
C3—H3	0.9500	C16—H16A	0.9800
C3—C4	1.348 (14)	C16—H16B	0.9800
C15—C1	1.406 (14)	C16—H16C	0.9800
			
O7—S1—C17	104.0 (5)	C8—C7—H7	120.0
O7—S1—C16	107.9 (6)	C6—C7—H7	120.0
C17—S1—C16	95.7 (6)	O3—C4—C3	121.6 (9)
C4—O3—C15	121.0 (8)	O3—C4—C5	112.5 (9)
C8—O2—H2	109.5	C3—C4—C5	125.9 (9)
C9—O1—H1	109.5	C5—C10—H10	119.9
C13—O6—H6	109.5	C9—C10—C5	120.2 (10)
C11—O4—H4	109.5	C9—C10—H10	119.9
O2—C8—C9	114.6 (9)	C15—C14—H14	121.6
O2—C8—C7	125.5 (9)	C15—C14—C13	116.8 (9)
C7—C8—C9	119.9 (9)	C13—C14—H14	121.6
O5—C2—C3	122.2 (9)	O4—C11—C1	118.7 (10)
O5—C2—C1	121.9 (9)	O4—C11—C12	120.6 (10)
C3—C2—C1	115.9 (9)	C12—C11—C1	120.7 (10)
C2—C3—H3	119.0	O6—C13—C14	116.8 (9)
C4—C3—C2	122.0 (10)	O6—C13—C12	122.2 (10)
C4—C3—H3	119.0	C12—C13—C14	121.0 (11)
O3—C15—C1	119.3 (9)	S1—C17—H17A	109.5
C14—C15—O3	116.4 (9)	S1—C17—H17B	109.5
C14—C15—C1	124.3 (9)	S1—C17—H17C	109.5
C15—C1—C2	120.2 (9)	H17A—C17—H17B	109.5
C15—C1—C11	116.5 (10)	H17A—C17—H17C	109.5
C11—C1—C2	123.3 (9)	H17B—C17—H17C	109.5
C6—C5—C4	120.9 (9)	C11—C12—C13	120.6 (11)
C6—C5—C10	119.0 (9)	C11—C12—H12	119.7
C10—C5—C4	120.1 (9)	C13—C12—H12	119.7
C5—C6—H6A	119.7	S1—C16—H16A	109.5
C5—C6—C7	120.5 (9)	S1—C16—H16B	109.5
C7—C6—H6A	119.7	S1—C16—H16C	109.5
O1—C9—C8	115.9 (9)	H16A—C16—H16B	109.5
O1—C9—C10	123.7 (10)	H16A—C16—H16C	109.5
C10—C9—C8	120.4 (10)	H16B—C16—H16C	109.5
C8—C7—C6	120.0 (10)		
			
O3—C15—C1—C2	1.4 (15)	C15—C14—C13—O6	−178.5 (9)
O3—C15—C1—C11	−177.7 (9)	C15—C14—C13—C12	2.8 (17)
O3—C15—C14—C13	176.4 (10)	C1—C2—C3—C4	−0.6 (15)
O5—C2—C3—C4	179.0 (9)	C1—C15—C14—C13	−2.8 (16)
O5—C2—C1—C15	179.3 (10)	C1—C11—C12—C13	0.4 (17)
O5—C2—C1—C11	−1.8 (16)	C5—C6—C7—C8	0.1 (15)
O2—C8—C9—O1	−2.9 (13)	C6—C5—C4—O3	177.7 (9)
O2—C8—C9—C10	178.6 (10)	C6—C5—C4—C3	−1.8 (16)
O2—C8—C7—C6	−178.6 (10)	C6—C5—C10—C9	0.8 (15)
O1—C9—C10—C5	−178.3 (9)	C9—C8—C7—C6	0.7 (15)
O6—C13—C12—C11	179.6 (10)	C7—C8—C9—O1	177.7 (9)
O4—C11—C12—C13	179.4 (11)	C7—C8—C9—C10	−0.8 (15)
C8—C9—C10—C5	0.0 (15)	C4—O3—C15—C1	0.1 (14)
C2—C3—C4—O3	2.1 (16)	C4—O3—C15—C14	−179.1 (9)
C2—C3—C4—C5	−178.4 (10)	C4—C5—C6—C7	179.5 (10)
C2—C1—C11—O4	1.7 (16)	C4—C5—C10—C9	−179.6 (10)
C2—C1—C11—C12	−179.2 (10)	C10—C5—C6—C7	−0.9 (15)
C3—C2—C1—C15	−1.1 (14)	C10—C5—C4—O3	−1.9 (14)
C3—C2—C1—C11	177.8 (10)	C10—C5—C4—C3	178.6 (10)
C15—O3—C4—C3	−1.9 (14)	C14—C15—C1—C2	−179.5 (10)
C15—O3—C4—C5	178.6 (8)	C14—C15—C1—C11	1.5 (16)
C15—C1—C11—O4	−179.3 (9)	C14—C13—C12—C11	−1.7 (18)
C15—C1—C11—C12	−0.2 (16)		

### 3',4',5,7-Tetrahydroxyflavone dimethyl sulfoxide monosolvate Hydrogen-bond geometry (Å, º)

**Table:**

*D*—H···*A*	*D*—H	H···*A*	*D*···*A*	*D*—H···*A*
O1—H1···O5^i^	0.84	1.83	2.649 (11)	163
O2—H2···O1^ii^	0.84	2.14	2.846 (11)	142
O2—H2···O2^ii^	0.84	2.35	2.989 (11)	133
O4—H4···O5	0.84	1.79	2.626 (10)	170
O6—H6···O7^iii^	0.84	1.79	2.626 (10)	170
C3—H3···O1^iv^	0.95	2.54	3.220 (13)	129
C10—H10···O3	0.95	2.35	2.682 (11)	100
C16—H16*C*···O4^iii^	0.98	2.44	3.293 (15)	145
C17—H17*A*···O7^v^	0.98	2.39	3.308 (18)	156

Symmetry codes: (i) *x*+1, *y*−1, *z*; (ii) −*x*+3, *y*+1/2, −*z*+2; (iii) −*x*, *y*−1/2, −*z*+1; (iv) *x*−1, *y*+1, *z*; (v) −*x*, *y*+1/2, −*z*+1.
